# Supplementary material for: Near-Complete Genome of SARS-CoV-2 Delta (AY.3) Variant Identified in a Dog in Kansas, USA
Source: Viruses. 2021 Oct 19;13(10):2104. doi: 10.3390/v13102104 (PMC8541569; doi:10.3390/v13102104)
Supplement: Supplementary file 1 [file viruses-13-02104-s001.zip › viruses-1410293-supplementary.pdf]

Virus Name	GISAID #
hCoV-19/USA/KS-KSU-2046/2021	EPI_ISL_3693315
hCoV-19/USA/KS-KSU-1953/2021	EPI_ISL_3231270
hCoV-19/USA/KS-KSU-1982/2021	EPI_ISL_3693311
hCoV-19/USA/KS-KSU-2001/2021	EPI_ISL_3693312
hCoV-19/USA/KS-KSU-2015/2021	EPI_ISL_3693313
hCoV-19/USA/KS-KSU-2016/2021	EPI_ISL_3693314
hCoV-19/USA/KS-KSU-2047/2021	EPI_ISL_3693316
hCoV-19/USA/KS-KSU-2048/2021	EPI_ISL_3693317
hCoV-19/USA/KS-KSU-2067/2021	EPI_ISL_3693318
hCoV-19/USA/KS-KSU-2068/2021	EPI_ISL_3693319
hCoV-19/USA/KS-KSU-2136/2021	EPI_ISL_3693320
hCoV-19/USA/KS-KSU-2146/2021	EPI_ISL_3693321
hCoV-19/USA/KS-KSU-2180/2021	EPI_ISL_3941711
hCoV-19/USA/KS-KSU-2267/2021	EPI_ISL_3941712
hCoV-19/USA/KS-KSU-2305/2021	EPI_ISL_3941713
hCoV-19/USA/KS-KSU-2302/2021	EPI_ISL_3941714
hCoV-19/USA/KS-KSU-2325/2021	EPI_ISL_3941715
hCoV-19/USA/KS-KSU-2402/2021	EPI_ISL_3941716
hCoV-19/USA/KS-KSU-2509/2021	EPI_ISL_3941717
hCoV-19/USA/KS-KSU-2510/2021	EPI_ISL_3941718
hCoV-19/USA/KS-KSU-2511/2021	EPI_ISL_3941719
hCoV-19/USA/KS-KSU-2512/2021	EPI_ISL_3941720
hCoV-19/USA/KS-KSU-2601/2021	EPI_ISL_3941721
hCoV-19/USA/KS-KSU-2671/2021	EPI_ISL_3941722
hCoV-19/USA/KS-KSU-2672/2021	EPI_ISL_3941723
hCoV-19/USA/KS-KSU-2731/2021	EPI_ISL_3941724
hCoV-19/USA/KS-KSU-2812/2021	EPI_ISL_3941725
hCoV-19/USA/KS-KSU-2882/2021	EPI_ISL_3941726
hCoV-19/USA/KS-KSU-2938/2021	EPI_ISL_3941727
hCoV-19/USA/KS-KSU-2183/2021	EPI_ISL_3941728

Supplemental Table 1. SARS-CoV-2 sequences utilized in this study.

Coding region	Nucleotide site	Nucleotide change	Amino acid change
**5'UTR**	210	G>T	-
	241	C>T	-
**ORF 1ab**	3037	C>T	-
	4181	G>T	A>S
	6402	C>T	P>L
	7124	C>T	P>S
	8986	C>T	-
	9053	G>T	V>L
	10029	C>T	T>I
	11201	A>G	T>A
	11333	A>G	-
	14408	C>T	P>L
	15451	C>A	G>S
	16467	C>T	P>L
	19220	C>T	A>V
**S**	21618	C>G	T>R
	22911	T>G	L>R
	22989	C>A	T>K
	23397	A>G	D>G
	23598	C>G	P>R
	24404	G>A	D>N
**3a**	25497	C>T	S>L
**E**	26761	T>C	I>T
**7ab**	27632	T>C	V>A
	27746	C>T	T>I
**8**	27868	C>T	T>I
**N**	28448	A>G	D>G R>M
	28868	G>T	
	28903	G>T	G>L
	29389	G>T	D>Y
**3'UTR**	29729	G>T	-

Supplemental Table 2. SARS-CoV-2 Kansas genomic changes. Nucleotide and amino acid changes are indicated.
